# The joint involvement in adult onset Still's disease is characterised by a peculiar magnetic resonance imaging and a specific transcriptomic profile

**DOI:** 10.1038/s41598-021-91613-5

**Published:** 2021-06-14

**Authors:** Piero Ruscitti, Antonio Barile, Onorina Berardicurti, Sonia Iafrate, Paola Di Benedetto, Antonio Vitale, Francesco Caso, Luisa Costa, Federico Bruno, Francesco Ursini, Luca Navarini, Federica Sensini, Raffaele Scarpa, Bruno Frediani, Luca Cantarini, Carlo Masciocchi, Roberto Giacomelli, Paola Cipriani

**Affiliations:** 1 Department of Biotechnological and Applied Clinical Sciences, University of L’Aquila, L’Aquila, Italy; 2 Research Center of Systemic Autoinflammatory Diseases and Behçet’s Disease Clinic, Department of Medical Sciences, Surgery and Neurosciences, University of Siena, Siena, Italy; 3 Rheumatology Unit, Department of Clinical Medicine and Surgery, University Federico II of Naples, Naples, Italy; 4 Department of Biomedical and Neuromotor Sciences (DIBINEM), Alma Mater Studiorum University of Bologna, Bologna, Italy; 5 Medicine and Rheumatology Unit, IRCCS Istituto Ortopedico Rizzoli, Bologna, Italy; 6 Unit of Allergology, Immunology, Rheumatology, Department of Medicine, University of Campus Bio-Medico of Rome, Rome, Italy

**Keywords:** Pathogenesis, Rheumatology

## Abstract

Adult onset Still's disease (AOSD) is a rare systemic autoinflammatory disease, characterised by fever, arthritis, and skin rash, and joint involvement is one of its clinical manifestations. The aims of this work were to assess joint involvement, to describe main patterns of involvement, and associated clinical characteristics. In this work, we aimed at assessing the joint involvement in AOSD by using MRI, to describe main patterns and associated clinical characteristics. In addition, we aimed at assessing the global transcriptomic profile of synovial tissues in AOSD to elucidate possible pathogenic pathways involved. We also evaluated the global transcriptomic profile of synovial tissues to elucidate possible pathogenic pathways involved in the disease. Thus, AOSD patients, who underwent to MRI exam on joints, were assessed to describe patterns of joint involvement and associated clinical characteristics. Some synovial tissues were collected for RNA-sequencing purposes. The most common MRI finding was the presence of synovitis on 60.5%, mainly in peripheral affected joints, with low to intermediate signal intensity on T1-weighted images and intermediate to high signal intensity on T2-fat-saturated weighted and STIR images. Bone oedema and MRI-bone erosions were reported on 34.9% and 25.6% MRI exams, respectively. Patients with MRI-bone erosions showed a higher prevalence of splenomegaly, a more frequent chronic disease course, lower levels of erythrocyte sedimentation rate, and ferritin. In AOSD synovial tissues, a hyper-expression of interleukin (IL)-1, IL-6, and TNF pathways was shown together with ferritin genes. In conclusion, in AOSD patients, the most common MRI-finding was the presence of synovitis, characterised by intermediate to high signal intensity on T2-fat-saturated weighted and STIR images. MRI-bone erosions and bone oedema were also observed. In AOSD synovial tissues, IL-1, IL-6, and TNF pathways together with ferritin genes resulted to be hyper-expressed.

## Introduction

Adult-onset Still’s disease (AOSD) is a rare inflammatory disease of unknown aetiology^1^. These patients are constantly affected by fever, which starts suddenly with daily spikes. Arthritis, either oligoarthritis or bilateral symmetrical rheumatoid arthritis-like polyarthritis, is another common clinical feature of AOSD^2^. The joint involvement may have with a migrating pattern at the beginning and it may become becoming stable over the time^2–4^. Although any joint may be affected, wrists, knees and ankles are those more frequently involved affected^5^. During the early phases of AOSD or flare, conventional radiography may display normal or a soft tissue swelling in association with a mild periarticular demineralisation of involved affected joints^6^. Subsequently, patients may develop a distinctive pattern of intercarpal and carpometacarpal joint space narrowing, leading to joint erosions and/or pericapitate ankylosis^7,8^. The latter is considered a classic and suggestive feature of AOSD^7–9^. AOSD patients with chronic course may show a distinctive pattern of intercarpal and carpometacarpal joint space narrowing, leading to joint erosions and/or pericapitate ankyloses^7,8^. The latter may be considered a classic *sequela* in the chronic pattern of the disease^7–9^. In this context, the role of magnetic resonance imaging (MRI) in AOSD patients is not elucidated yet.

MRI is a non-ionizing imaging technique with an excellent soft-tissue visualisation. It allows a multiplanar tomographic imaging, without a projectional superimposition and possible geometric distortions associated with other techniques^10^. Furthermore, inflammatory soft tissue changes of synovitis, which are not detectable by conventional clinical assessment and conventional radiography biochemical, and radiographic methods, may be directly visualised and evaluated in detail by MRI^11^. In addition, MRI this technique offers a large anatomical coverage and has the possibility of standardised image acquisition and possible centralised reading^12,13^. Importantly, MRI is a very safe technique, without involving no ionising radiations and without increasing the risk of malignancies^10–13^. However, during AOSD, a systematic evaluation of joint involvement by MRI is still missing. On these bases, in this work, we aimed at assessing the joint involvement in AOSD by using MRI, to describe main patterns and associated clinical characteristics. In addition, we aimed at assessing the global transcriptomic profile of synovial tissues in AOSD to elucidate possible pathogenic pathways involved in the disease with.

## Results

### Patients and descriptive characteristics

In this study, 31 patients with AOSD (mean age 42.3 ± 15.2 years, 54.8% male gender), who underwent to at least one MRI exam on joints, were assessed. The clinical characteristics were registered at the time of MRI execution. The most common clinical manifestations were fever (87.1%), arthralgia (80.6%), and arthritis (77.4%). Systemic score resulted to be 4.2 ± 2.4 (mean ± SD). Inflammatory markers were also increased during flares of the disease [erythrocyte sedimentation rate (ESR): 49.7 ± 16.5 mm/h, C-reactive protein (CRP): 50 (90) mg/L median (interquartile range), ferritin: 1757.7 (3975.0) ng/mL]. A large percentage of these patients were treated with low dosage of glucocorticoids (GCs) (54.8%), synthetic- (51.6%), and biologic-disease modifying anti rheumatic drugs (DMARDs) (54.8%). Specifically, 7 patients were treated with tocilizumab, 5 with anakinra, 4 with etanercept, and 1 with infliximab. No episode of macrophage activation syndrome (MAS) was reported in this cohort. Supplementary Table 1 summarizes descriptive statistics of assessed patients.

### MRI findings in AOSD

**Figure 1:**
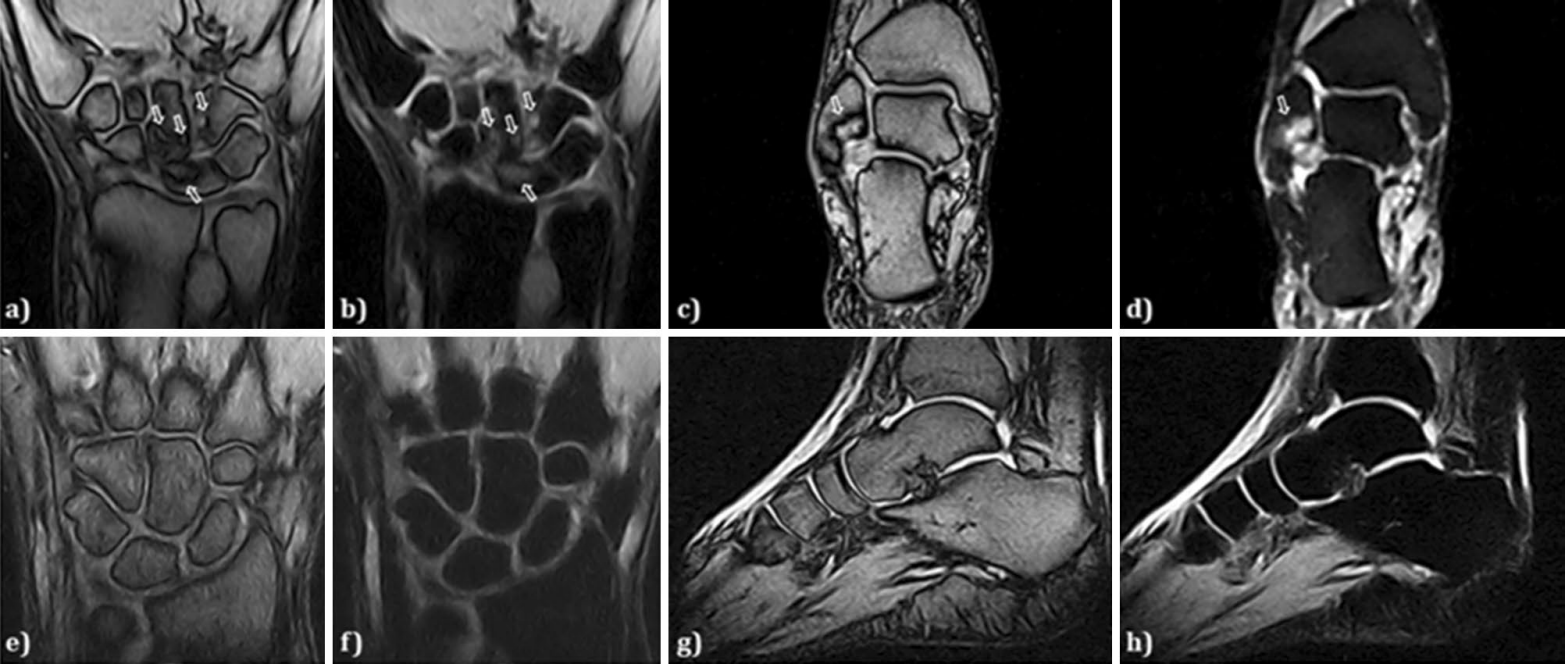
Synovitis, bone oedema, and bone erosions, as common MRI findings on wrists and ankles in AOSD. In panels (**a**) and (**b**), T1-weighted and T2-STIR images of coronal sections on wrist are shown, respectively, detailing a mild to moderate proliferative synovitis, as thickening of the synovial membrane, with low to intermediate signal intensity on T1-weighted images and intermediate to high signal intensity on T2-STIR images (arrows). Bone oedema and bone erosions are also shown, which overlapped the locations (arrows). In panel (**c**) and (**d**), T1-weighted and T2-STIR images of coronal sections on ankle are shown, respectively, detailing a mild to moderate proliferative synovitis, with low to intermediate signal intensity on T1-weighted images and intermediate to high signal intensity on T2-STIR images (arrows). Bone oedema and bone erosion are also shown, which overlapped the locations (arrows). In panels (**e**) and (**f**) T1-weighted and T2-fat saturated images of coronal sections on wrist are shown, respectively, detailing no pathologic findings. In panels (**c**) and (**d**), T1-weighted and T2-STIR images of sagittal sections on ankle are shown, respectively, detailing no pathologic findings.

The present evaluation included 44 MRI exams (13 exams of hand/wrists, 12 of knee, 5 of pelvis, 3 of shoulder, 3 of ankle, 3 of feet, 2 of hip, 2 of lumbosacral spine and 1 of cervical spine). The most common MRI finding was the presence of synovitis on 26 (60.5%) exams, mainly in peripheral affected joints, as shown in Fig. 1. In these exams, MRI revealed a mild to moderate proliferative synovitis, as thickening of the synovial membrane, suggesting the presence of a hyperplastic than of a hypertrophied synovial tissue. In these patients, we observed a low to intermediate signal intensity on T1-weighted images and an intermediate to high signal intensity on T2-fat saturated weighted and STIR images. Contrast enhancement (CE)-MRI was performed in our series in 20 patients with severe synovial involvement, allowing to better differentiate active hyperplastic synovitis than fluid effusion. CE-MRI confirmed the findings on T2-fat saturated weighted and STIR images without CE, as shown in Supplementary Figure 1. The presence of bone oedema, characterised by high signal intensity on T2-fat saturated weighted and STIR images, was reported on 15 (34.9%) MRI exams. In 11 (25.6%) patients, MRI-bone erosions were observed, codified as on T1-weigheted images as discontinuity of signal void of cortical bone and loss of normal high signal intensity of bone marrow fat. In all patients but one, MRI-bone erosions were synchronous with bone oedema, overlapping completely the locations, as reported in Fig. 1. Less frequently, tenosynovitis, defined as peritendinous effusion, and a focal joint space narrowing were reported on 8 (18.6%) and 7 (16.3%) patients MRI exams, respectively. Concerning MRI on spine, although the low number, no inflammatory lesions were detected. No ankylosis was observed in any MRI, even if this technique could not be the first choice in assessing this feature.

### Follow-up study

**Figure 2:**
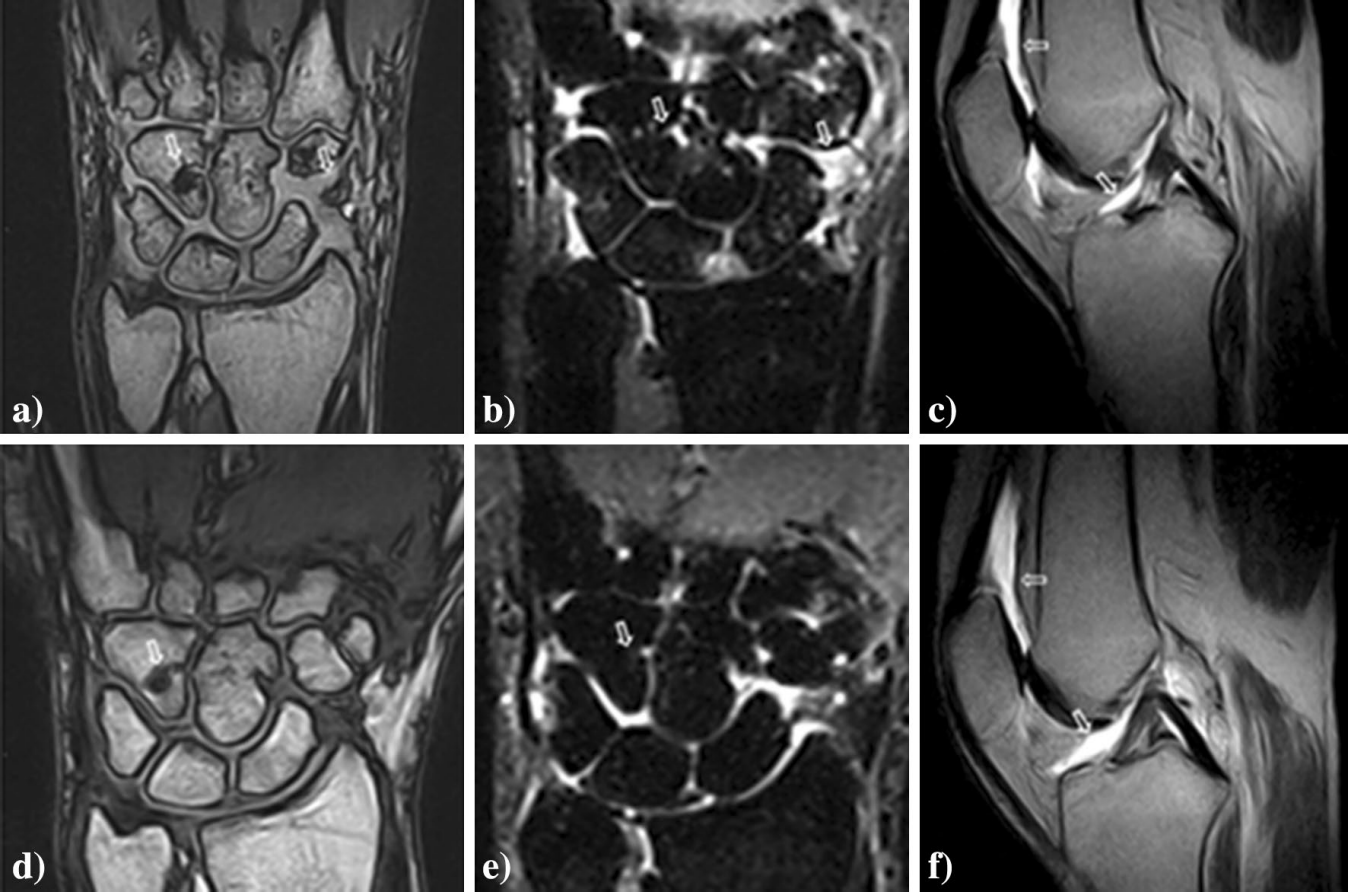
MRI findings before and after therapies in AOSD. In panels (**a**) and (**b**), T1-weighted and T2-STIR images of coronal sections on wrist are shown, respectively, before therapies (**a**,**b**), whereas, in panels (**d**) and (**e**), T1-weighted and T2-STIR images of coronal sections on wrist are shown, after therapies, reporting the reduction of synovitis, the disappearance of bone oedema, and the stabilisation of bone erosions (**d**,**e**) (arrows). In panels (**c**) and (**f**), T2-weighted images of sagittal sections on knee are shown before (**c**) and after therapies (**f**), respectively, detailing a worsening of proliferative synovitis and effusion in suprapatellar bursa and intra-articular space (arrows).

During the follow-up, in 20 patients, 20 MRI exams were repeated (6 of hand/wrist, 5 of knee, 4 of pelvis, 2 of ankle, 1 of hip, 1 of cervical spine and 1of lumbar spine), and evaluated for possible changes, without any inferential analysis due to the lack of power. Following the therapies, an improvement of synovitis and bone oedema was reported, whereas a stabilisation of MRI-bone erosions. Conversely, a worsening of synovitis was reported on MRI exam during a flare of the disease, as shown in Fig. 2. On two MRI exams of spine, no changes were described during the follow-up.

### Patients with MRI bone-erosions, clinical characteristics, and predictive factors

Patients with and without MRI bone-erosions were grouped and compared, as reported in Supplementary Table 1. Patients with MRI-bone erosions showed a higher prevalence of splenomegaly [9 (90.0%) vs 11 (52.4%), p = 0.041], lower levels of ESR [46.6 ± 17.3 mm/h vs 52.5 ± 14.9 mm/h, p = 0.002], and ferritin [1191.7 (2288.5) ng/mL vs 2833.8 (3887.1) ng/mL, p = 0.044]. Furthermore, these patients were treated more frequently with both synthetic- [8 (80.0%) vs 8 (38.1%), p = 0.029] and biologic-DMARDs [8 (80.0%) vs 9 (42.8%), p = 0.046] experiencing more often a chronic disease course [7 (70.0%) vs 2 (9.5%), p = 0.007]. We performed the same analysis about disease courses grouping the patients according to the presence or not of synovitis. Non-significant results were retrieved about polycyclic [synovitis: 10 (50%) vs no-synovitis 5 (45.4%), p = 0.210] and chronic patterns [synovitis: 4 (20.0%) vs no-synovitis 5 (45.4%), p = 0.298]. In addition, possible correlations among the presence of MRI-bone erosions and ESR (coefficient − 0.123, p = 0.216), CRP (coefficient − 0.046, p = 0.385), ferritin (coefficient 0.15, p = 0.370), and systemic score (coefficient 0.88, p = 0.287) were searched, but non-significant results were obtained. Finally, a multivariate regression model was built to evaluate a possible predictive role of selected variables (use of synthetic DMARDs, chronic disease course) on the likelihood of MRI-bone erosions, assessing the first available MRI exam. The multivariate analysis showed that the use of synthetic DMARDs (OR 7.79, 95% CI 1.29–46.89, p = 0.025) and the chronic disease course (OR 1.69, 95% CI 1.04–28.43, p = 0.034) were independently related with MRI-bone erosions, as reported in Table 1. The logistic regression model was statistically significant (χ^2^ = 10.99, p = 0.001).

**Table 1:** Multivariate regression analysis of predictive factors on the likelihood of MRI-bone erosions in AOSD.

Clinical variables	OR	SE	95% CI	p
**Univariate analyses**				
Age	1.02	0.02	0.97–1.07	0.352
Male gender	2.25	0.72	0.55–9.24	0.261
Fever	1.03	1.21	0.09–11.12	0.978
Arthritis	2.80	1.12	0.30–25.77	0.363
Skin rash	0.75	0.80	0.15–3.59	0.715
Splenomegaly	6.01	1.11	0.68–12.90	0.107
Systemic score	1.09	0.16	0.81–1.48	0.565
Disease duration	0.98	0.08	0.85–1.14	0.865
ESR	0.99	0.35	0.49–1.99	0.989
CRP	0.91	0.24	0.56–1.44	0.666
Ferritin	1.44	0.26	0.85–2.42	0.168
Low dosage of GCs	0.50	0.70	0.12–1.99	0.327
Synthetic DMARDs	7.50	0.86	1.38–40.69	**0.002**
Biologic DMARDs	4.56	0.86	0.98–10.75	0.061
Polycyclic disease pattern	0.83	0.72	0.20–3.44	0.803
Chronic disease pattern	5.20	0.75	1.18–22.89	**0.029**
**Multivariate analysis**				
Synthetic DMARDs	7.79	0.92	1.29–46.89	**0.025**
Chronic disease pattern	1.69	0.84	1.04–28.43	**0.034**
Constant χ^2^ = 10.99, p = **0.001**				

*MRI* magnetic resonance imaging, *AOSD* adult onset Still’s disease, *OR* odds ratio, *SE* standard error, *95% CI* 95% confidence interval, *ESR* erythrocyte sedimentation rate, *CRP* C-reactive protein, *GCs* glucocorticoids, *DMARDs* disease modifying anti-rheumatic drugs. Bolded values are statistically significant (p < 0.05).

### Histological findings

During the follow-up, in two patients, replacements of hip were performed and the synovial tissues were collected. Based on the peculiar MRI signal intensity of synovitis, we conducted a pathogenic study on these synovial samples. In this evaluation, we performed a histological study to evaluate the pro-inflammatory infiltrate and the presence of some cytokines involved in AOSD pathogenesis. Two patients (female 25 years-old, male 54 years-old, respectively) underwent joint surgery since a repeated and uncontrolled inflammatory involvement of hips because of the disease. They reported a long history of AOSD, treated with glucocorticoids, 0.2–0.3 mg/kg/day of prednisone equivalent, and non-steroidal anti-inflammatory drugs, on-demand, during the acute flares. In these patients, at the time of synovial tissues collection, no biologic DMARD was administered.

As shown in Supplementary Figure 2, a moderate perivascular mononuclear infiltrate in the sub-lining stroma of hip synovial tissues was observed, associated with a slight increase in the cellular density of the stroma and a synovial lining of 2–3 layers. These features could suggest a low-grade synovitis. Furthermore, an increase number of vessels number could be suggested, but without a haematic extravasation. In addition, interleukin (IL)-1β, IL-6, TNF, and heavy ferritin subunit (FeH) were found on AOSD synovial tissues, as shown in Supplementary Figure 3. Finally, CD68+/FeH+ cells were observed in these tissues, as reported in Supplementary Figure 4.

### RNA sequencing analysis

**Figure 3:**
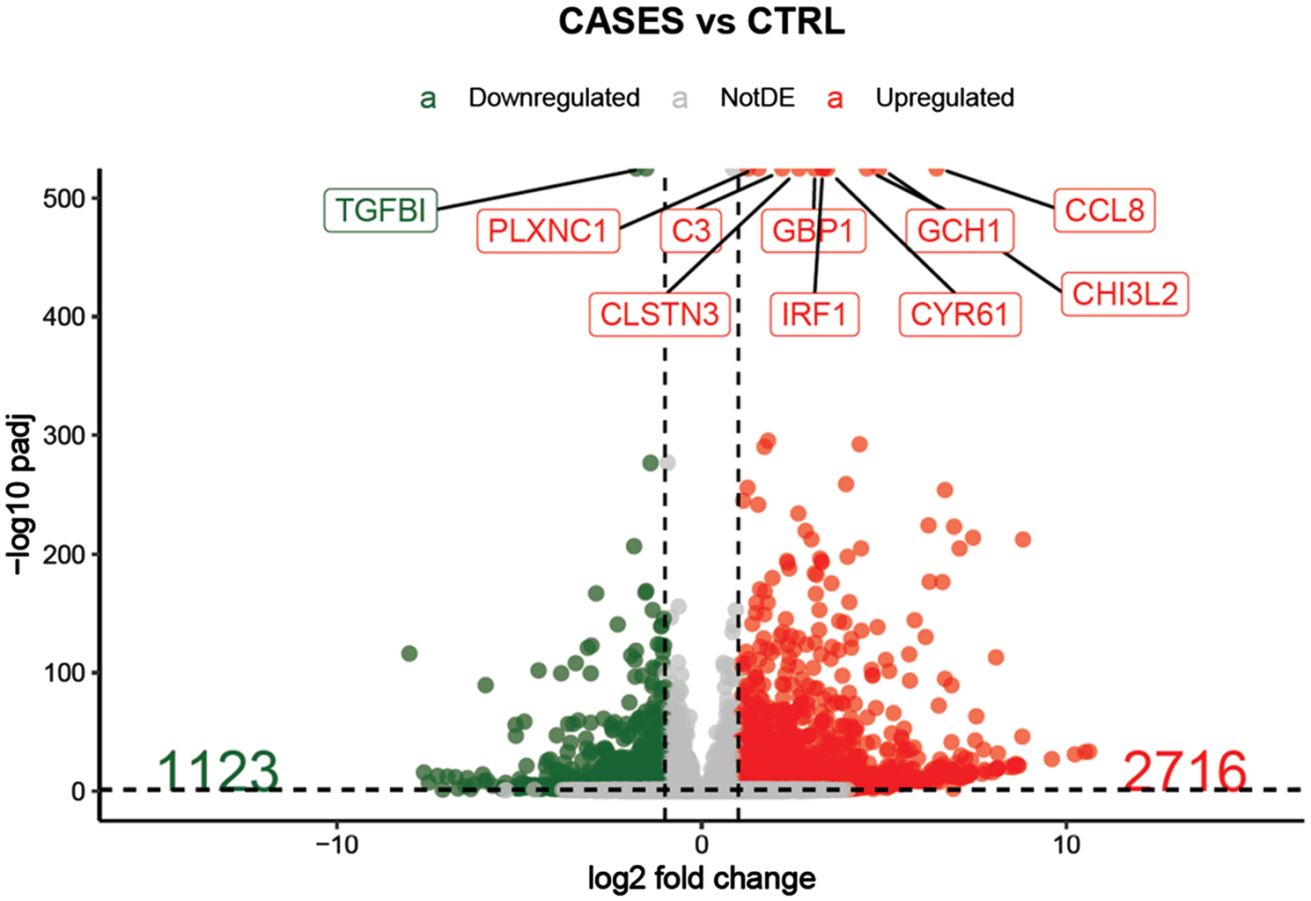
Volcano plot showing the effect size log2 fold-change (x axis) and significance (y axis) of each tested gene. Cut-off: log2FC ≥ 1 or log2FC ≤ − 1 and FDR < 0.05.

**Figure 4:**
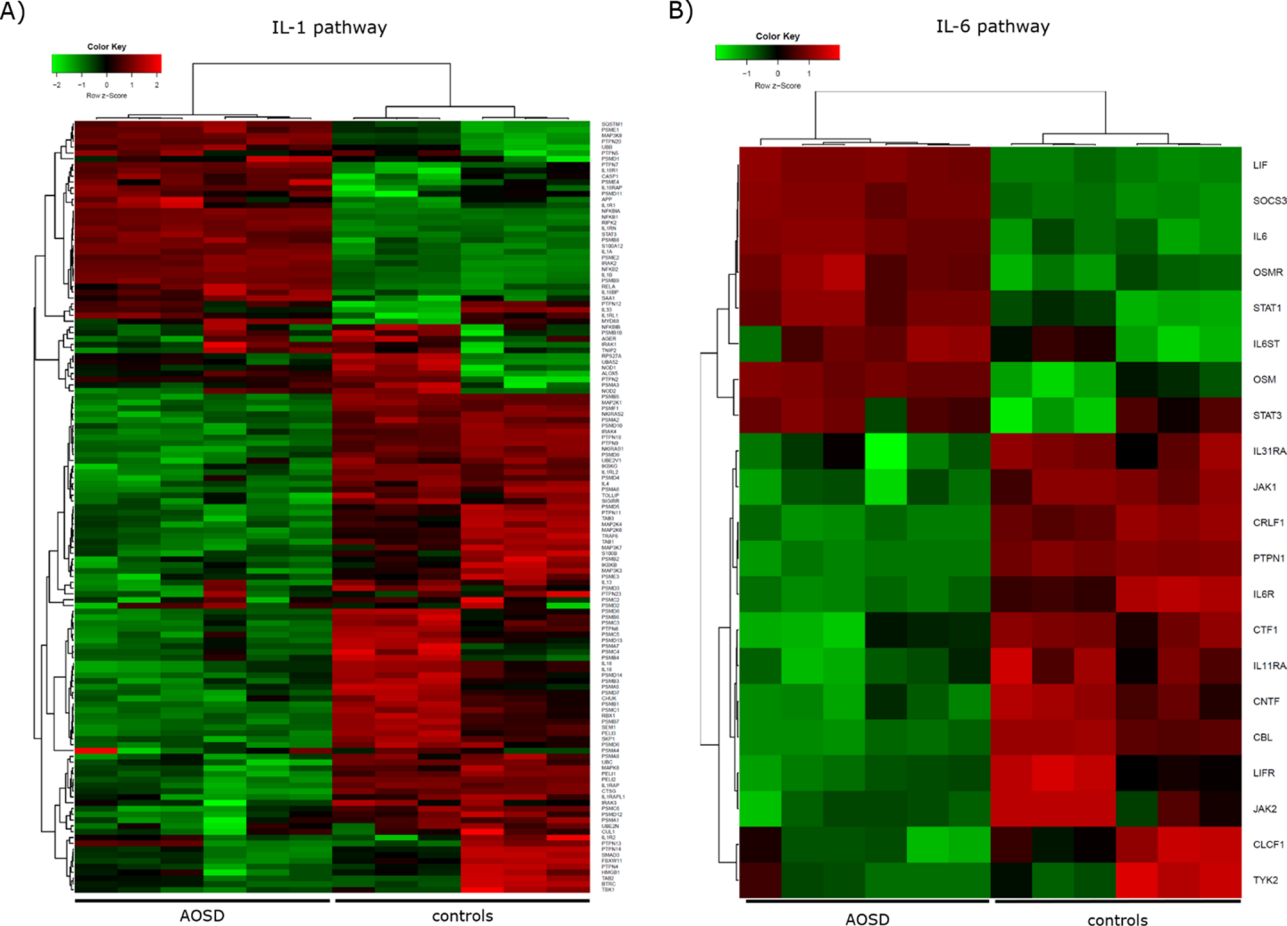
Clustered heatmaps of IL-1 (**A**) and IL-6 (**B**) pathways (R package version 1.0.0, https://CRAN.R-project.org/package=ggplot.multistats).

**Figure 5:**
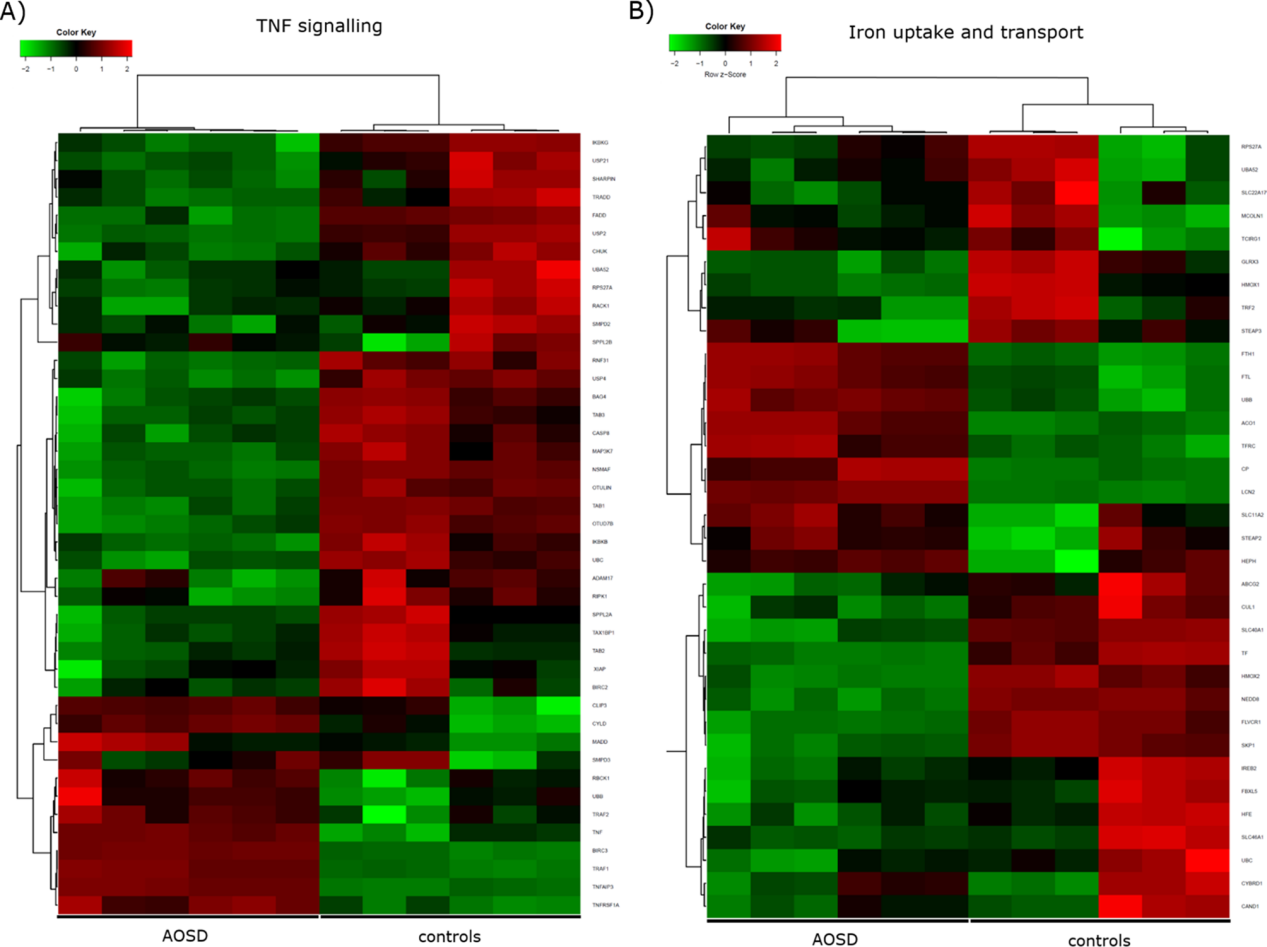
Clustered heatmaps of TNF signalling (**A**), and iron uptake and transport pathways (**B**) (R package version 1.0.0, https://CRAN.R-project.org/package=ggplot.multistats).

After the histological assessment, we performed in the same patients a bulky RNA-sequencing to provide a transcriptomic synovial tissue profile of main pathogenic pathways involved in AOSD. This, RNA-sequencing analysis assessed the global transcriptomic profile of synovial tissues on AOSD patients and gender matched-controls (female 70 years-old, male 65 years-old, respectively). The comparison of the gene expressions profile between AOSD patients and controls revealed 1123 genes downregulated (log2FC ≤ − 1 and FDR < 0.05) and 2716 genes upregulated (log2FC ≥ 1 and FDR < 0.05) in AOSD synovial tissues (Fig. 3). Among upregulated ones, *plxnc1*, *clstn3*, *c3*, *gbp1*, *irf1*, *gch1*, *cyr61*, *ccl8*, and *chi3l2* showed the highest differences between the 2 groups (Fig. 3). After that, we explored genes implicated in IL-1, IL-6, TNF, IFN-γ, and iron uptake and transport pathways. Assessing IL-1 pathway, we found an increased expression of *il1a*, *il1b*, *il1rap*, *il1r1*, *il18r1*, and *il18bp* on AOSD tissues when compared with controls. On the contrary, *il18* resulted to be more expressed in the controls (Fig. 4A). In IL-6 pathway, we found an increased expression of *il6* and *il6st/gp130* on AOSD synovial tissues, whereas an increased expression of *il6r* was shown in the controls (Fig. 4B). Among genes involved in TNF pathway, *tnf*, *traf1*, *traf2*, *tnfaip3*, and *tnfrsf1a* resulted to be more expressed in AOSD synovial tissues than controls (Fig. 5A). Furthermore, no significant difference was retrieved analysing the interferon (IFN)-γ pathway (Supplementary Figure 5). Finally, *fth1* and *ftl* were more expressed in AOSD patients than controls (Fig. 5B), when we explored the iron uptake and transport pathway.

## Discussion

The present work showed MRI patterns of joint involvement in AOSD, mainly reporting the presence of synovitis, characterised by low to intermediate signal intensity on T1-weighted images and intermediate to high signal intensity on T2-fat saturated weighted and STIR images. MRI-bone erosions and bone oedema in peripheral joints were also observed, which overlapped the locations. Assessing these findings with clinical features, the subset of patients showing MRI-bone erosions was characterised by a chronic disease course, lower levels of both ferritin and ESR. In addition, a global transcriptomic profile of synovial tissues suggested specific pathogenic pathways involved in AOSD joint involvement.

The most common MRI finding was the presence of synovitis in peripheral joints, including wrists, shoulders, and knees, which showed a low to intermediate signal intensity on T1-weighted images and an intermediate to high signal intensity on T2-fat saturated weighted and STIR images. The latter feature appears to differentiate AOSD synovitis from haemorrhagic or post-traumatic ones, which are typically characterised by low or high intensity on T2-fat saturated weighted and STIR images, respectively^14,15^. This feature could be associated with the presence of FeH on AOSD synovial tissues, which could represent a specific marker of the disease, as observed in other affected tissues^16,17^. Despite the lack of power, the results obtained from the follow-up, suggested that MRI-synovitis could also follow the activity of the disease, reducing or enhancing in case of remission or flare. Thus, MRI-synovitis could be considered a possible surrogate of disease activity in AOSD to be assessed during the disease course, at least regarding the joint involvement.

In addition, a percentage of patients showed the presence of MRI-bone erosions and bone oedema. These findings seemed to be synchronous between them, overlapping the locations, and it could suggesting another typical feature of AOSD joint involvement. Differently from what previously reported^6–9^, no MRI findings to be correlated with ankylosis were observed on MRI-exams. In fact, although considered a typical feature of AOSD, the pericapitate ankylosis on conventional radiograph may be considered a finding of a long-standing and insufficiently treated disease. Thus, our MRI exams could have not revealed this aspect since the characteristics of assessed patients, with a relatively low disease duration and all variably treated with glucocorticoids (GCs), synthetic- and/or biologic DMARDs.

Analysing comparatively clinical characteristics and MRI-findings, the results suggested some clinical features related to MRI-bone erosions. In fact, these patients with MRI-bone erosions were characterised by a higher prevalence of splenomegaly, lower levels of both ESR and ferritin, and experienced more commonly a chronic disease pattern. In this context, it has been reported that splenomegaly and chronic disease pattern could predict the need of prolonged therapies^18^, differently from what observed in patients achieving a monocyclic pattern^19^. In fact, these patients were more frequently treated with synthetic- and biologic-DMARDs, which are administered in non-responder patients to first-line therapeutic strategies^20,21^.

However, analysing predictive factors of MRI-bone erosions, the use of synthetic-DMARDs was predictive of this finding, whereas the use of biologic-DMARDs did not. In fact, During AOSD, the administration of biologic-DMARDs is related to additional prescribing motivations to joint involvement in AOSD, considering the heterogeneous clinical course of these patients the disease^22^. The association among MRI-bone erosions and lower levels of both ESR and ferritin could reflect a more intensive therapeutic strategy in these patients, paralleling what observed in rheumatoid arthritis as a confounding by indication bias^23,24^. Another possible explanation could be provided by a recent published multidimensional characterisation of the disease AOSD patients^25^. In this work, four different clusters of AOSD patients were identified by using values of systemic score, ferritin, CRP, ESR, and age. Patients codified in cluster 4 were characterised by lower levels of ferritin and ESR than others. Thus, it could be possible that patients with MRI-erosions in the present evaluation could be codified in this cluster 4. These observations may suggest the need of further studies to fully validate the proposed clinical clusters of AOSD and to evaluate associated clinical characteristics.

Interestingly, the global transcriptomic profile of AOSD synovial tissues suggested the hyper-expression of IL-1α, IL-1β, IL-6, and TNF as well as of their respective receptors, furtherly reinforcing the rationale of inhibiting these cytokines during the disease^26^. Thus, it could be possible to speculate the existence of a specific pro-inflammatory signature on AOSD joint involvement, characterised by the hyper-activation of these pathways. In addition, the hyper-expression of ferritin genes on AOSD synovial tissues could highlight the idea of a possible pathogenic role of ferritin in enhancing the pro-inflammatory burden, as proposed by the concept of the “hyper-ferritinaemic syndrome”^27–29^.

Finally, AOSD is burdened by life-threatening complications, such as MAS mainly MAS, a hyper-inflammatory syndrome which leads to a high mortality rate, although the disease usually affects young people^30–32^. Interestingly, in our cohort, no episode of MAS was registered. In our study, the patients in our cohort did not appear to be characterised by a hyper-activation of IFN-γ pathway, which is considered of crucial importance on the occurrence of life-threatening complications during AOSD and its juvenile counterpart^33–36^. In fact, the activation of IFN-γ would prompt the occurrence of a systemic inflammation, since it has been implicated as a pivotal mediator of the pathogenesis in different hyper-inflammatory diseases^35,36^. Similarly, although elevated levels of IL-18 are were reported in AOSD^37^, this cytokine appeared downregulated in synovial tissues of our AOSD patients. This possible discrepancy could be related to the pathogenic role of IL-18 in development of both MAS and cytokine storm syndrome^38,39^. Thus, it could be possible that IL-18 could be also more involved in the systemic manifestations of the disease than the articular ones. In addition, considering that synovial tissues were collected in patients with a long history of AOSD, the assessed cells could be in a state in which the production of IL-18 could be reduced due to the drugs and/or the exhaustion of pro-inflammatory cells producing cytokines. Taking together these observations, further studies are needed to fully evaluate these issues.

The present evaluation is affected by different limitations due to the observational study design and the lack of control group, reducing the validity of our findings and suggesting a cautious generalization of the results. Furthermore, the relative low number of assessed MRI-exams would recommend the necessity of specific designed studies to entirely clarify this issue. However, the rarity of AOSD makes very difficult to arrange prospective studies, suggesting the significance of cohort studies to identify relevant clinical features and to generate hypotheses to be subsequently investigated.

In conclusion, in this work, MRI patterns of AOSD joint involvement were reported suggesting possible peculiar features on these patients. The most common finding was the presence of synovitis, characterised by an intermediate to high signal intensity on T2-fat saturated weighted and STIR images. MRI-bone erosions and bone oedema in peripheral joints were also observed, which could be synchronous between them, overlapping the locations. Assessing these findings with clinical features, the subset of patients showing MRI-bone erosions was characterised by a chronic disease course, lower levels of both ESR and ferritin. In addition, a hyper-activation was observed on of IL-1, IL-6, TNF, and ferritin pathways were observed by a global transcriptomic profile of AOSD synovial tissues, suggesting pathogenic mechanisms and therapeutic targets. Taking together all these findings, although further studies are needed to entirely elucidate these issues, some specific aspects of AOSD could be observed on joint involvement, suggesting typical radiologic findings, associated clinical features, involved pathogenic mechanisms, and therapeutic targets.

## Methods

### Study design

From January 2015 to December 2019, consecutive patients with AOSD, who underwent at least one MRI exam on joints, were assessed, in an observational study, to describe patterns of joint involvement by using MRI and to highlight associated clinical characteristics and predictive factors.

The local Ethics Committee (Comitato Etico Azienda Sanitaria Locale 1 Avezzano/Sulmona/L’Aquila, L’Aquila, Italy) approved the study, considering both clinical (protocol number 0139815/16) and translational (protocol number 0122353/17) sections of the present research project. The study was performed according to the Good Clinical Practice guidelines and the Declaration of Helsinki. Each patient provided the informed consent for purposes of the study.

### Settings

Patients with AOSD, who attended the Rheumatologic Clinics of L’Aquila, Siena, Naples, and Bologna, were considered. These Italian Rheumatology centres, involved in this project, were selected due to previous expertise on management of AOSD and in inception cohort studies.

### Patients

Patients were considered whether fulfilling Yamaguchi’s criteria^40^ for diagnosis of AOSD and undergoing at least one MRI exam on joints during the follow-up, for a clinical suspicion of joint involvement by the disease. Since the observational nature of the study, the choice of performing or not the MRI of joint was left to the physicians in charge on the patient. After the execution of this radiologic exam, the data of these patients were registered.

### Variables on MRI exams

MRI exam was included if performed as detailed in available imaging protocols in musculoskeletal system^41^. The strict adherence to these standardised protocols has been deemed as crucial to minimise the variability among different diagnostic centres and readers, involved in the present study. In cases with severe synovitis, CE-MRI was employed to a better characterisation of the findings, by a bolus of gadolinium (0.1 mmol/kg). MRI-findings, namely bone erosions, osteitis/bone marrow oedema, synovitis, joint space narrowing, and tenosynovitis, were scored, by experienced radiologists, according to OMERACT MRI definitions^42^.

### Clinical variables

In this work, we related clinical characteristics and predictive factors of joint involvement in AOSD. These findings were recorded at the time of MRI execution. Clinical features, systemic score, occurrence life-threatening complications, therapies, and patterns of disease were reported. The systemic score was derived, as previously detailed^6,43^. The presence of fever, arthritis, arthralgia, complications, and other clinical manifestations were also evaluated and defined as discretely distributed variables (yes/no). The occurrence of MAS and other AOSD-life threatening complications were evaluated as previously reported^32,44^. Patients in remission were defined as those achieving a complete disappearance of any clinical and laboratory feature of the disease as previously reported^43^. Inflammatory markers, ESR and CRP, and ferritin were registered, and expressed as continuous variables. At the end of follow-up, patients were codified into three different disease courses, monocyclic, polycyclic, and chronic patterns as previously performed^6,32,43–46^. The administration of therapies for managing AOSD was also recorded, at the diagnosis and during the follow-up, GCs, synthetic- and biologic-DMARDs. The use of GCs was stratified into two categories, low dosage (≤ 0.5 mg/kg/day of prednisone-equivalent) and high dosage (> 0.5 mg/kg/day of prednisone-equivalent), as already done^43^.

### Data sources

Relevant data were collected at study beginning and reassessed, during the scheduled visits for each involved participant by an extensive clinical history. A specific form was used to retrieve all variables of interest for study purposes.

### Bias

Considering the observational design, our study could be subjected to a number of possible biases, which we tried to overcome by a relatively simple study design and a careful selection of involved centres, all to be considered tertiary referral centres for AOSD and with a high experience in inception cohort studies. We also tried to minimise the main methodological issues by a careful definition of each variable to be assessed. Furthermore, participants with significant missing data, which were considered to be meaningful for the analyses, were removed.

### Study size

No sample size estimation was provided, since our evaluation would provide a “real-life” estimation of the joint patterns of involvement in AOSD. From January 1, 2015 to December 31, 2019, consecutive patients with AOSD, admitted to Italian Rheumatology Units, who underwent to at least 1 MRI exam on the joint, were assessed and followed-up.

### Hematoxylin and eosin

During the follow-up, in two patients, replacements of hip were performed and the synovial tissues were collected for histological purposes. Paraffin-embedded hip synovial tissues were processed for haematoxylin and eosin (H&E) staining. Samples were analysed at a single cutting level according to standard of practice. Synovial sections were analysed according to a semi-quantitative assessment of synovitis according to a previously validated score^47^. Images were acquired with Olympus BX53 microscope.

### Immunofluorescence

Immunofluorescence analysis was performed on paraffin sections (thickness 3 μm). The sections were dewaxed, and antigen retrieval was carried out using Dako Target Retrieval solution (DAKO, USA). After blocking, with Dako Protein block (Dako, USA), the sections were incubated for 60 min at room temperature with the following antibodies anti-CD68 (sc-9139, 1:20, Santa Cruz Biotechnology, USA), anti-FeH (sc-376594, 1:50, Santa Cruz Biotechnology, USA). The immunoreaction was revealed using conjugated secondary antibody (Alexa Fluor; Life Technologies, USA). Negative controls were obtained by omitting the primary antibody. Cell nuclei were visualized using 4′,6-diamidino-2-phenylindole. Fluorescence was acquired using a BX53 fluorescence microscope (Olympus, Center Valley, PA, USA).

### Immunohistochemistry

Synovia paraffin sections (thickness 3 μm) were deparaffinised, treated with endogenous peroxidase blocking (Dako, USA) and then with Dako Protein block (Dako, USA) to block non-specific binding. After blocking, sections were incubated respectively with anti-IL-1 (sc-7884, 1:100, Santa Cruz Biotechnology, USA), anti-IL-6 (sc-130326, 1:100, Santa Cruz Biotechnology, USA), anti-TNFα (sc-8301, Santa Cruz Biotechnology, USA) and anti-FeH antibody (sc-376594, 1:200, Santa Cruz Biotechnology, USA). Visualisation of the primary antibodies was performed using EnVision Flex/HRP and DAB (diaminobenzidine) (both Dako, USA). No immunohistochemical staining was noted in negative control samples where the primary antibody was omitted. Sections were examined and photographed under light microscope (Olympus BX53).

### RNA sequencing of synovial tissues

Total RNA was extracted from synovial tissue using All prep DNA/RNA/miRNA universal kit (Qiagen, Germany) As controls, we used 2 patients with hip fracture who did a hip arthroplasty. Extracted RNA was submitted to an external company (GALSEQ srl, Via Vincenzo Monti, 8, 20123 Milano) for RNA sequencing, including total RNA sample detection, mRNA enrichment, synthesis of double-stranded cDNAs, end repair/dA-tailing module, fragment selection, PCR amplification, library detection, and Illumina sequencing. The method for screening differentially expressed genes between 2 groups was filtered by P value of less than 0.05, and fold-change (FC) values were more than 1.2-fold. Heatmaps were generated in R: a Language and Environment for Statistical Computing statistical software (version 3.0.3; R core team, R Foundation for Statistical Computing, Vienna, Austria, 2018, https://www.R-project.org/) with the heatmap.2 function in the ggplots (R package version 1.0.0, https://CRAN.R-project.org/package=ggplot.multistats).

### Statistical methods

Statistics firstly provided the descriptive analysis of all assessed variables, in included patients. The data distribution was tested by using Shapiro–Wilk test and, thus, collected results were presented as mean and standard deviation (SD) or median and interquartile range (IQR). To compare the clinical characteristics of patients with- and without MRI-erosions parametric or non-parametric *t* tests were used for all the continuous variables and Chi-squared test was used for the categorical variables, as appropriate. Furthermore, possible correlations among the presence of MRI-bone erosions and ESR, CRP, ferritin, and systemic score were estimated by using a point-biserial coefficient correlation. In addition, a regression analysis was built to evaluate possible predictive factors, assessing the significance of selected clinical and laboratory features, on the likelihood of MRI-erosions presence. The statistical analysis of this feature was planned considering that the variable was discretely distributed (“yes MRI-bone erosions”/”no MRI-bone erosions”). The selection process of covariates started by a univariate analysis of each variable, any variable having a significant univariate test was selected as candidate for the multivariate analysis, whereas removed if non-significant. At the end of this process, the multivariate model was provided, with OR estimations of independently predictors of MRI-bone erosions. Due to the relatively simple study design, a very few missing data were managed by exclusion of these from analyses. Two-sided P values < 0.05 were considered as being statistically significant. The Statistics Package for Social Sciences (SPSS for Windows, version 17.0, SPSS Inc., Chicago, IL, USA) was used for all analyses.

### Ethics approval and consent to participate

The local Ethics Committee (Comitato Etico Azienda Sanitaria Locale 1 Avezzano/Sulmona/L’Aquila, L’Aquila, Italy; protocol number 0139815/16 and protocol number 0122353/17) approved the study, which was performed according to the Good Clinical Practice guidelines and the Declaration of Helsinki.

Informed consent was obtained from each patient for the use of de-identified clinical and laboratory data and images for study purposes.

## Supplementary Information

Supplementary Information.

## Data Availability

All data relevant to the study are included in the article or uploaded as supplementary information.

## Abbreviations

AOSD: Adult-onset Still’s disease
MRI: Magnetic resonance imaging
MAS: Macrophage activation syndrome
ESR: Erythrocyte sedimentation rate
CRP: C-reactive protein
GCs: Glucocorticoids
DMARDs: Disease modifying anti-rheumatic drugs
H&E: Haematoxylin and eosin
SD: Standard deviation
IQR: Interquartile range
