# Supplementary material for: The joint involvement in adult onset Still's disease is characterised by a peculiar magnetic resonance imaging and a specific transcriptomic profile
Source: Sci Rep. 2021 Jun 14;11:12455. doi: 10.1038/s41598-021-91613-5 (PMC8203668; doi:10.1038/s41598-021-91613-5)
Supplement: Supplementary file 1 — Supplementary Information. [file 41598_2021_91613_MOESM1_ESM.docx]

**Title Page supplementary materials**

-Full Title:

The joint involvement in adult onset Still's disease is characterised by peculiar magnetic resonance imaging and a specific transcriptomic profile

-Complete given names and surnames of authors:

Piero Ruscitti^1◊^*, Antonio Barile^1◊^,Onorina Berardicurti^1◊^,Sonia Iafrate^1^,Paola Di Benedetto^1^,Antonio Vitale^2^,Francesco Caso^3^, Luisa Costa^3^,Federico Bruno^1^, Francesco Ursini^4,5^, Luca Navarini^6^, Federica Sensini^1^, Raffaele Scarpa^3^, Bruno Frediani^2^, Luca Cantarini^2^, Carlo Masciocchi^1^,Roberto Giacomelli^6§^,Paola Cipriani^1§^.

^◊^: contributed equally to this work

^§^: contributed equally to this work

*-*Key indexing terms:

Adult onset Still’s disease, magnetic resonance imaging, pathogenesis.

-Affiliations:

^1^: Department of Biotechnological and Applied Clinical Sciences, University of L'Aquila, L'Aquila, Italy;

^2^: Research Center of Systemic Autoinflammatory Diseases and Behçet's Disease Clinic, Department of Medical Sciences, Surgery and Neurosciences, University of Siena, Siena, Italy;

^3^: Rheumatology Unit, Department of Clinical Medicine and Surgery, University Federico II of Naples, Naples, Italy;

^4^: Department of Biomedical and Neuromotor Sciences (DIBINEM), Alma Mater Studiorum University of Bologna, Bologna, Italy;

^5^: Medicine & Rheumatology Unit, IRCCS Istituto Ortopedico Rizzoli, Bologna, Italy;

^6^: Unit of Allergology, Immunology, Rheumatology, Department of Medicine, University of Campus Bio-Medico of Rome, Rome, Italy.

- Name and address of author responsible for correspondence:

PieroRuscitti MD PhD; Rheumatology Unit; Department of Biotechnological and Applied Clinical Sciences, University of L'Aquila; delta 6 building, L'Aquila, PO box 67100; Italy; e-mail addresses: pieroruscitti@live.com piero.ruscitti@univaq.it; fax number +390862433523; telephone number +390862434742.

- A short running head title:

MRI in AOSD

STROBE checklist pag.4

Supplementary Table 1 pag.6

Supplementary Figure 1 pag.8

Supplementary Figure 2 pag.10

Supplementary Figure 3 pag.12

Supplementary Figure 4 pag.13

Supplementary Figure 5 pag.14

**STROBE Statement—Checklist of items that should be included in reports of *cohort studies***

|  | Item No | Recommendation | Page No |
| --- | --- | --- | --- |
| **Title and abstract** | 1 | (*a*) Indicate the study’s design with a commonly used term in the title or the abstract | 1 |
|  |  | (*b*) Provide in the abstract an informative and balanced summary of what was done and what was found | 3 |
| Introduction | | | |
| Background/rationale | 2 | Explain the scientific background and rationale for the investigation being reported | 4 |
| Objectives | 3 | State specific objectives, including any prespecified hypotheses | 4 |
| Methods | | | |
| Study design | 4 | Present key elements of study design early in the paper | 5 |
| Setting | 5 | Describe the setting, locations, and relevant dates, including periods of recruitment, exposure, follow-up, and data collection | 5 |
| Participants | 6 | (*a*) Give the eligibility criteria, and the sources and methods of selection of participants. Describe methods of follow-up | 5 |
|  |  | (*b*)For matched studies, give matching criteria and number of exposed and unexposed |  |
| Variables | 7 | Clearly define all outcomes, exposures, predictors, potential confounders, and effect modifiers. Give diagnostic criteria, if applicable | 5 |
| Data sources/ measurement | 8* | For each variable of interest, give sources of data and details of methods of assessment (measurement). Describe comparability of assessment methods if there is more than one group | 5 |
| Bias | 9 | Describe any efforts to address potential sources of bias | 5 |
| Study size | 10 | Explain how the study size was arrived at |  |
| Quantitative variables | 11 | Explain how quantitative variables were handled in the analyses. If applicable, describe which groupings were chosen and why | 5 |
| Statistical methods | 12 | (*a*) Describe all statistical methods, including those used to control for confounding | 5 |
|  |  | (*b*) Describe any methods used to examine subgroups and interactions | 5 |
|  |  | (*c*) Explain how missing data were addressed | 5 |
|  |  | (*d*) If applicable, explain how loss to follow-up was addressed | 5 |
|  |  | (*e*) Describe any sensitivity analyses | 5 |
| Results | | |  |
| Participants | 13* | (a) Report numbers of individuals at each stage of study—eg numbers potentially eligible, examined for eligibility, confirmed eligible, included in the study, completing follow-up, and analysed | 6 |
|  |  | (b) Give reasons for non-participation at each stage | 6 |
|  |  | (c) Consider use of a flow diagram |  |
| Descriptive data | 14* | (a) Give characteristics of study participants (eg demographic, clinical, social) and information on exposures and potential confounders | 6 |
|  |  | (b) Indicate number of participants with missing data for each variable of interest |  |
|  |  | (c) Summarise follow-up time (eg, average and total amount) |  |
| Outcome data | 15* | Report numbers of outcome events or summary measures over time | 6 |

| Main results | 16 | (*a*) Give unadjusted estimates and, if applicable, confounder-adjusted estimates and their precision (eg, 95% confidence interval). Make clear which confounders were adjusted for and why they were included | 6 |
| --- | --- | --- | --- |
|  |  | (*b*) Report category boundaries when continuous variables were categorized |  |
|  |  | (*c*) If relevant, consider translating estimates of relative risk into absolute risk for a meaningful time period |  |
| Other analyses | 17 | Report other analyses done—eg analyses of subgroups and interactions, and sensitivity analyses | 6 |
| Discussion | | | |
| Key results | 18 | Summarise key results with reference to study objectives | 11 |
| Limitations | 19 | Discuss limitations of the study, taking into account sources of potential bias or imprecision. Discuss both direction and magnitude of any potential bias | 13 |
| Interpretation | 20 | Give a cautious overall interpretation of results considering objectives, limitations, multiplicity of analyses, results from similar studies, and other relevant evidence | 13 |
| Generalisability | 21 | Discuss the generalisability (external validity) of the study results | 13 |
| Other information | | | |
| Funding | 22 | Give the source of funding and the role of the funders for the present study and, if applicable, for the original study on which the present article is based |  |

*Give information separately for exposed and unexposed groups.

**Note:** An Explanation and Elaboration article discusses each checklist item and gives methodological background and published examples of transparent reporting. The STROBE checklist is best used in conjunction with this article (freely available on the Web sites of PLoS Medicine at http://www.plosmedicine.org/, Annals of Internal Medicine at http://www.annals.org/, and Epidemiology at http://www.epidem.com/). Information on the STROBE Initiative is available at http://www.strobe-statement.org.

**Supplementary Table 1. Descriptive statistics of clinical characteristics of assessed patients with AOSD, grouped according to the presence of MRI-bone erosions.**

| **AOSD Descriptive characteristics** | | | | |
| --- | --- | --- | --- | --- |
|  | *31 patients* | *10 patients with MRI-erosions* | *21 patients without MRI-erosions* | *P values,*  *Patients with MRI-erosions vs patients without MRI-erosions* |
| *Clinical characteristics* |  |  |  |  |
| Age, mean ± SD | 42.3 ± 15.2 years | 46.9 ± 13.9 years | 40.1 ± 15.6 years | 0.680 |
| Gender, n (%) | 17 (54.8) male | 7 (70.0) male | 10 (47.6) | 0.242 |
| Fever, n (%) | 27 (87.1) | 9 (90.0) | 18 (85.7) | 0.739 |
| Arthritis, n (%) | 24 (77.4) | 9 (90.0) | 15 (71.4) | 0.248 |
| Arthralgia, n (%) | 25 (80.6) | 8 (80.0) | 17 (80.9) | 0.950 |
| Skin Rash, n (%) | 21 (67.7) | 7 (70.0) | 14 (66.7) | 0.575 |
| Myalgia, n (%) | 20 (64.5) | 6 (60.0) | 14 (66.7) | 0.717 |
| Splenomegaly, n (%) | 20 (64.5) | 9 (90.0) | 11 (52.4) | **0.041** |
| Lymph node involvement, n (%) | 16 (51.6) | 6 (60.0) | 10 (47.6) | 0.519 |
| Sore throat, n (%) | 14 (45.2) | 6 (60.0) | 8 (38.1) | 0.167 |
| Liver Involvement, n (%) | 14 (45.2) | 4 (40.0) | 10 (47.6) | 0.252 |
| Pericarditis, n (%) | 6 (19.4) | 0 (0.0) | 6 (28.6) | 0.061 |
| Pleuritis, n (%) | 4 (12.9) | 0 (0.0) | 4 (19.1) | 0.139 |
| Pneumonia, n (%) | 3 (9.7) | 0 (0.0) | 3 (14.3) | 0.209 |
| Abdominal Pain, n (%) | 3 (9.7) | 1 (10.0) | 2 (9.5) | 0.096 |
| Disease duration, median (interquartile range) | 2.5 (15) years | 2.7 (14) years | 2.2 (15) years | 0.987 |
| Systemic score, mean ± SD | 4.2 ± 2.4 | 5.0 ± 2.2 | 4.1 ± 2.6 | 0.122 |
| *Complications* | | | | |
| MAS, n (%) | 0 (0.0) | 0 (0.0) | 0 (0.0) | / |
| *Laboratory* | | | | |
| Leucocytosis >15000/mm^3^, n (%) | 12 (38.7) | 2 (20.0) | 10 (47.6) | 0.140 |
| ESR, mean ± SD | 49.7 ± 16.5 mm/hr | 46.6 ± 17.3 mm/hr | 52.5 ± 14.9 mm/hr | **0.002** |
| CRP, median (interquartile range) | 50 (90) mg/L | 72 (89) mg/L | 83 (87) mg/L | 0.787 |
| Ferritin, median (interquartile range) | 1757.7 (3975.0) ng/mL | 1191.7 (2288.5) ng/mL | 2833.8 (3887.1) ng/mL | **0.044** |
| *Therapies* | | | | |
| Low dosage GCs, n (%) | 17 (54.8) | 5 (50.0) | 12 (57.1) | 0.709 |
| High Dosage GCs, n (%) | 11 (35.5) | 4(40.0) | 7 (33.3) | 0.717 |
| Synthetic DMARDs, n (%) | 16 (51.6) | 8 (80.0) | 8 (38.1) | **0.029** |
| Biologic DMARDs, n (%) | 17 (54.8) | 8 (80.0) | 9 (42.8) | **0.046** |
| *Disease patterns and follow-up* | | | | |
| Polycyclic disease pattern, n (%) | 15 (48.4) | 4 (40.0) | 11 (52.4) | 0.057 |
| Chronic disease pattern, n (%) | 9 (29.0) | 7 (70.0) | 2 (9.5) | **0.007** |
| Time of follow-up, median (interquartile range) | 4.4 (15) years | 4.3 (14.2) years | 4.8 (15.0) years | 0.869 |

MRI: magnetic resonance imaging; AOSD: adult onset Still’s disease; n: number of patients; DMARDs: disease-modifying anti-rheumatic drugs; MAS: macrophage activation syndrome; ESR: erythrocyte sedimentation rate; CRP: C-reactive protein; GCs: glucocorticoids. Bolded values are statistically significant (p < 0.05).

**Supplementary Figure 1. Synovitis as common MRI-pathogenic findings on knees.**

**
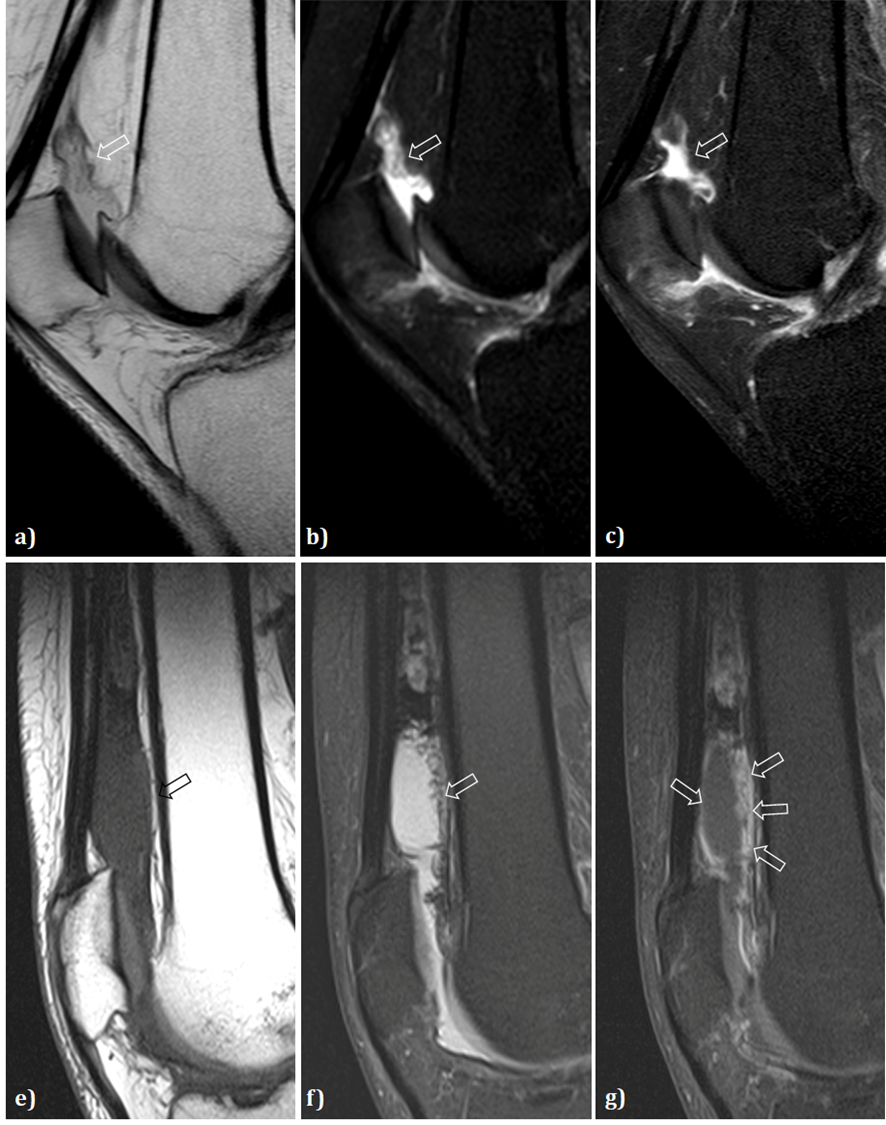
**

In panels a) and b), T2-weighted image of axial section andT2-weighted image of sagittal sectionon knee are shown, respectively, detailing a mild to moderate proliferative synovitis associated with effusion in *gastrocnemio-semimebranousus bursa*(arrows). In panel c) and d), T2-weighted images of sagittal sections of shoulders are shown, detailing a mild to moderate proliferative synovitis (arrows).

**Supplementary Figure 2. MRI pathogenic findings on hip in AOSD and associated histological findings.**

**
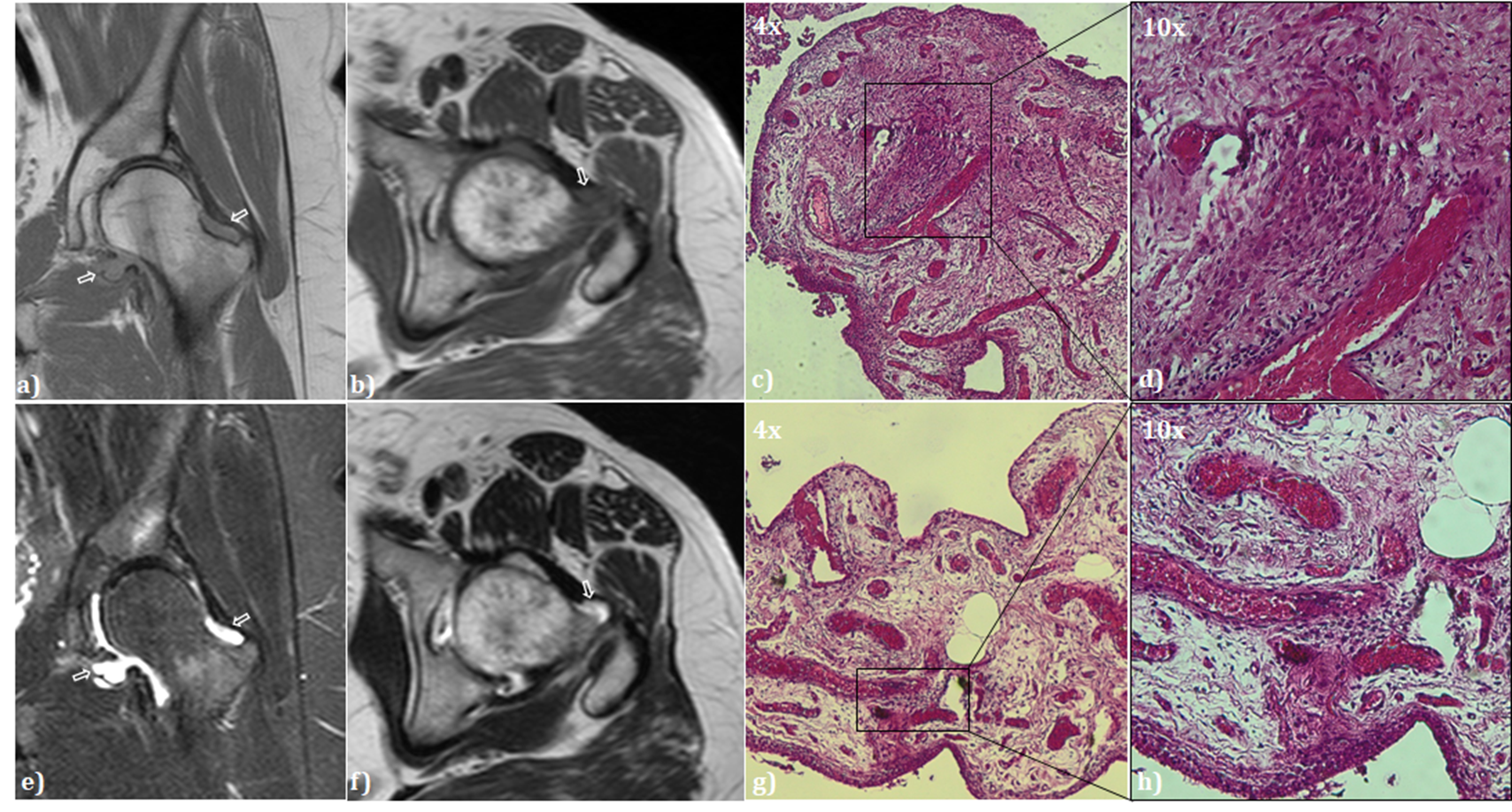
**

In panels a), b), e) and f), T1-weighted images of coronal (a) and axial (b) sections T2-STIR image of coronal section, and T2-weighted of axial section ankle, on hip are shown, respectively, detailing a moderate proliferative synovitis and effusion (arrows). In panels c), d), g) and h), original magnification 4X and 10X H&E stained tissues are shown a low-grade synovitis. A moderate perivascular mononuclear infiltrate in the sub-lining stroma is associated with a slight increase in the cellular density of the stroma and a synovial lining of 2-3 layers. Furthermore, an increase number of vessels number could be suggested, but without haematic extravasation.

**Supplementary Figure 3. Expression of IL-1β (A), IL-6 (B), TNF-α (C), and FeH subunit (D) in synovial liningcells and sublining.** Magnification 10x.


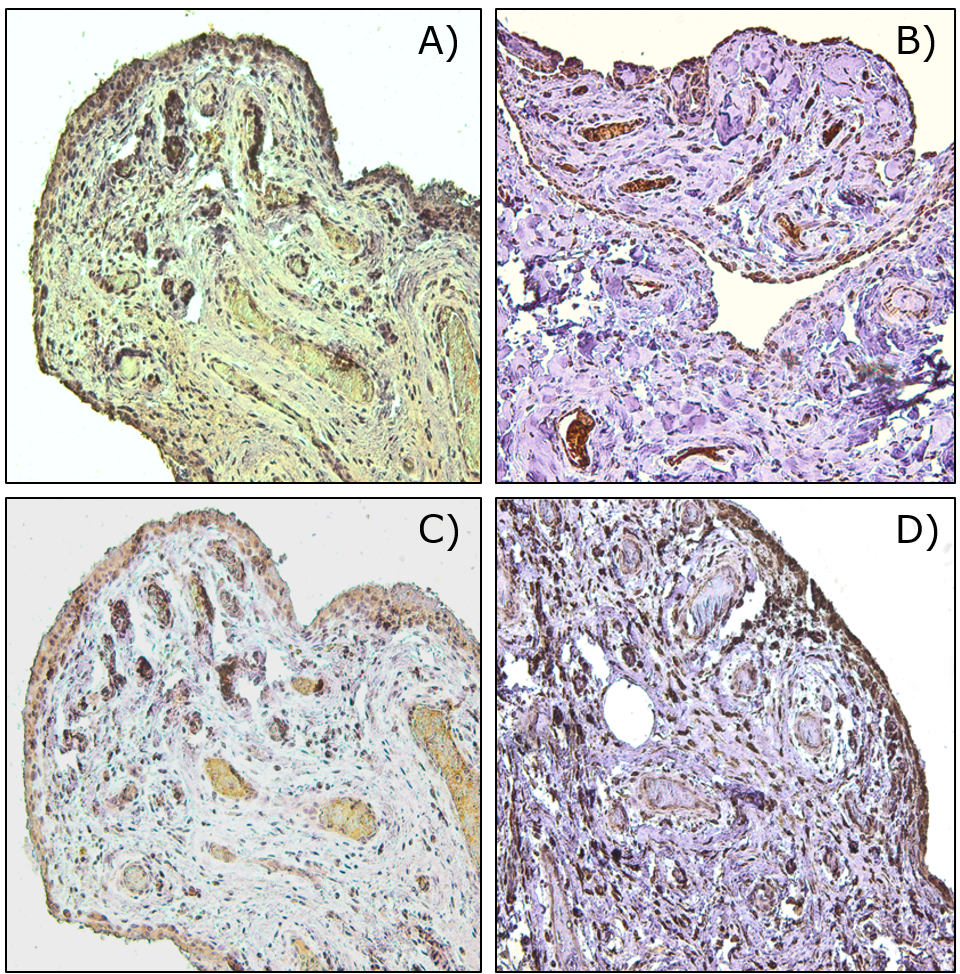


**Supplementary Figure 4 (A-B). Immunofluorescence showing CD68+ cells (red) and FeH subunit (green) in synovial sublining.** Magnification 10x. White arrows indicate CD68+ cells.


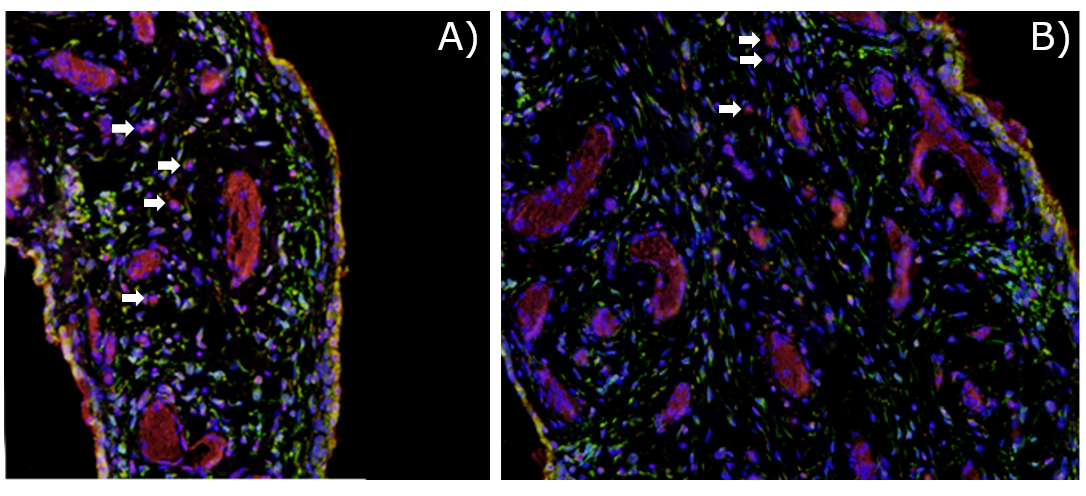


**Supplementary Figure 5. Clustered heatmap of IFN-γ signalling.**

**
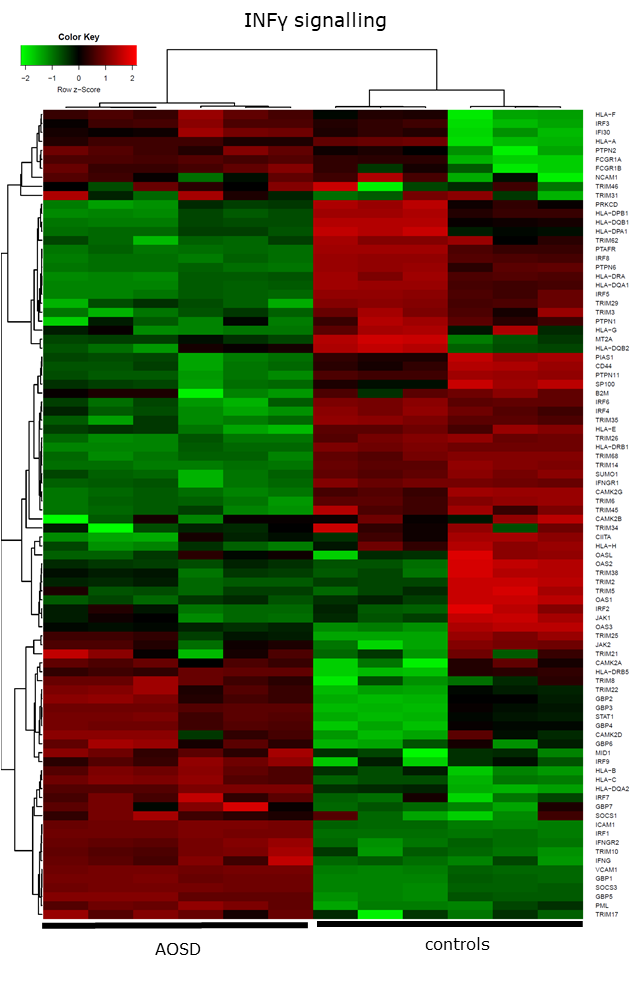
**

(R package version 1.0.0, https://CRAN.R-project.org/package=ggplot.multistats).
